# The heat shock protein family gene *Hspa1l* in male mice is dispensable for fertility

**DOI:** 10.7717/peerj.8702

**Published:** 2020-03-23

**Authors:** Xin Wang, Wenxiu Xie, Yejin Yao, Yunfei Zhu, Jianli Zhou, Yiqiang Cui, Xuejiang Guo, Yan Yuan, Zuomin Zhou, Mingxi Liu

**Affiliations:** 1 Department of Histology and Embryology, Nanjing Medical University, Nanjing, China; 2 Animal Core Facility, Nanjing Medical University, Nanjing, Jiangsu, China

**Keywords:** *HSPA1L*, Spermatogenesis, Male infertility, Gene knockout

## Abstract

**Background:**

Heat shock protein family A member 1 like (*Hspa1l*) is a member of the 70kD heat shock protein (*Hsp70*) family. HSPA1L is an ancient, evolutionarily conserved gene with a highly conserved domain structure. The gene is highly abundant and constitutively expressed in the mice testes. However, the role of *Hspa1l* in the testes has still not been elucidated.

**Methods:**

*Hspa1l*-mutant mice were generated using the CRISPR/Cas9 system. Histological and immunofluorescence staining were used to analyze the phenotypes of testis and epididymis. Apoptotic cells were detected through TUNEL assays. Fertility and sperm motilities were also tested. Quantitative RT-PCR was used for analyzing of candidate genes expression. Heat treatment was used to induce heat stress of the testis.

**Results:**

We successfully generated *Hspa1l* knockout mice. *Hspa1l*^-/-^ mice exhibited normal development and fertility. Further, *Hspa1l*^-/-^ mice shown no significant difference in spermatogenesis, the number of apoptotic cells in testes epididymal histology, sperm count and sperm motility from *Hspa1l*^+/+^ mice. Moreover, heat stress does not exacerbate the cell apoptosis in *Hspa1l*^-/-^ testes. These results revealed that HSPA1L is not essential for physiological spermatogenesis, nor is it involved in heat-induced stress responses, which provides a basis for further studies.

## Introduction

Infertility is a widespread issue that affects approximately 70 million people worldwide. Approximately 9% of couples are affected by infertility, with male infertility thought to play a role in 50% of infertile couples estimated by the World Health Organization (WHO) (Barratt et al., 2017). Male infertility attributed to a variety of reasons including: genetic factors such as mutations (Fainberg & Kashanian, 2019; Signore et al., 2019); diseases such as testicular cancer, cryptorchidism (Skakkebaek et al., 2016); unhealthy habits such as alcohol, tobacco and drug addiction (Sansone et al., 2018); environmental stress such as intense exposure to heat, pesticides, radiation, radioactivity and other harmful substances (Durairajanayagam, Agarwal & Ong, 2015; Kesari, Agarwal & Henkel, 2018). Spermatogenesis is a precisely regulated and complex process. Adult male testicular tissue consists of somatic and spermatogenic cells. Somatic cells in the testis mainly include Sertoli cells, Leydig cells, and testicular peritubular cells (TPCs). These somatic cells form the testicular microenvironment, and play an important role in regulating spermatogenesis. Spermatogenic cells mainly include spermatogonia (SPG), which are located in the basal compartment of seminiferous tubules. Human SPG can be divided into three different types: dark type A, light type A and B type SPG. Spermatogonial stem cells (SSCs) are self-renewing undifferentiated SPGs, and are thought to exist in type A SPGs (Fayomi & Orwig, 2018). Primary spermatocytes (SPCs) extend into the lumen of the seminiferous tubules, and are generated by SPGs through the transition process from mitosis to meiosis. SPCs can be divided into five stages: leptotene, zygotene, pachytene, diplotene and diakinesis. Primary SPCs undergo the first meiotic division to form secondary SPCs, then undergo a second meiosis to produce the round spermatids. These round spermatids further change shape, forming elongated spermatids that are then discharged into the lumen (Hermo et al., 2010; Neto et al., 2016; Wen et al., 2020). Germ cells development in the first wave of spermatogenesis in mice can be divided into the following stages: cells exhibiting stem-cell properties at postnatal day (PND) 0, spermatogonial mitosis at PND 7, pachyetene spermatocytes at PND 14, round spermatids at PND 20, and elongated spermatids at PND 28 (Chen et al., 2018b; Huang et al., 2008; Pence et al., 2019).

The HSP70 family is one of the most conserved protein families through evolution, from archaebacteria to plants and humans (Daugaard, Rohde & Jaattela, 2007; Radons, 2016). The HSP70s have a highly conserved domain structure containing a N-terminal nucleotide-binding domain (NBD) that exhibits ATPase activity, a middle flexible linker region, a substrate binding domain (SBD) that binds hydrophobic polypeptides, and an *α*-helical C-terminal domain (CTD) that seals the substrate and also mediates the binding of co-chaperone (Daugaard, Rohde & Jaattela, 2007; Radons, 2016; Vos et al., 2008; Wisniewska et al., 2010; Zuiderweg, Hightower & Gestwicki, 2017). The NBD and SBD always work together. The NBD binds and hydrolyzes ATP to regulate the affinity for substrates, and the binding of hydrophobic substrates to SBD accelerates the hydrolysis of ATP (Mayer, 2013; Mayer & Bukau, 2005).

Due to the ability of HSP70 to bind ATP and hydrophobic protein sequences, HSP70s play a crucial role in refolding denatured proteins, disaggregation, protein degradation, and maintaining protein homeostasis (Fernandez-Fernandez & Valpuesta, 2018). HSP70s also play a role in cell survival during stresses, including heat shock, oxidative stress, hypoxia, heavy metals, altered pH, inflammation, ischemia, fever, malignancy, etc. (Chen, Feder & Kang, 2018a; Ikwegbue et al., 2017; Radons, 2016; Singh et al., 2010). The changes in expression of HSP70s has been indicated in various  diseases, including cancer and nervous system diseases (Lee et al., 2001; Perez et al., 1991; Turturici, Sconzo & Geraci, 2011).

The human HSP70 family consists of 13 protein members which differ from each other in their amino acid sequences, subcellular locations and expression level (Daugaard, Rohde & Jaattela, 2007; Radons, 2016). In the HSP70 family, only HSPA2 and HSPA1L are mainly expressed in the testis. HSPA2 has been suggested to play an essential role in spermatogenesis, and is linked to male infertility (Dix et al., 1996; Erata et al., 2008; Nixon et al., 2015; Zhu, Dix & Eddy, 1997). *Hspa1l* is located in a major histocompatibility class (MHC) III cluster on 6p21.33 in human, where *Hspa1a* and *Hspa1b* are also located. *Hspa1a* and *Hspa1b* are more than 99% identical, and *Hspa1l* is 91% identical to *Hspa1a* (Daugaard, Rohde & Jaattela, 2007). HSPA1A and HSPA1B differ by only two amino acids. They are both stress-inducible and are functionally interchangeable so they are jointly named as HSP70 or HSP70-1 (Daugaard, Rohde & Jaattela, 2007; Radons, 2016). The basal mRNA expression of HSPA1A or HSPA1B varies in most tissues, and can be rapidly induced by stress (Murphy, 2013). The dysfunction of HSPA1A/B increases cells’ sensitivity to stress, and also affects the stability of vital organelles such as lysosomes and mitotic centrosomes (Dix, Garges & Hong, 1998; Fang et al., 2016; Huang, Mivechi & Moskophidis, 2001; Yamashima, 2012). Unlike *Hspa1a* and *Hspa1b*, *Hspa1l* is constitutively expressed, and is highly abundant in the testis (Daugaard, Rohde & Jaattela, 2007; Radons, 2016). The mutations of *Hspa1l* is supposed to be associated with inflammatory bowel disease, an increased risk of male infertility and spontaneous preterm births (Ciftci et al., 2015; Huusko et al., 2018; Kohan, Tabiee & Sepahi, 2019; Takahashi et al., 2017). HSPA1L has been thought to play important functions in testis similar to HSPA2 (Tsunekawa et al., 1999). However, the in vivo function of HSPA1L in testis has still not been elucidated in detail yet. Thus, in this study, we used the CRISPR/Cas9 system to generate *Hspa1l*-knockout mice to investigate the role of HSPA1L in testicular development and spermatogenesis.

## Materials & Methods

### Animals

Protocols for mice care, feeding, housing and treatments were drafted and implemented under the guidance of the Institutional Animal Care and Use Committee (IACUC) of Nanjing Medical University. The procedures of using and treating mice were approved by the Animal Ethical and Welfare Committee (Approval No. IACUC-1601117).

All mice were maintained under SPF conditions (12 h light/dark cycle, 20−26 °C, 50–55% humidity) with free access to water and food in Laboratory Animal Center of Nanjing Medical University. All mice were treated humanely and with efforts to minimize suffering. To induce loss of consciousness and death with a minimum of pain and distress, all mice were euthanized by cervical dislocation to collected tissue samples for further analyses. There were no surviving animals at the end of study.

### Generation of Hspa1l-mutant mice

We used the CRISPR/Cas9 technology to generate *Hspa1l*-knockout mice. The single-guide RNAs (sgRNAs) has been devised in the light of exon2 of *Hspa1l*. The sgRNA target sequences were 5′-CCACCAAGGATGCAGGTGTCATC-3 ′ and 5′- CGTGCACGAGTAGAAGCTGGACC-3 ′. The Cas9 plasmid and sgRNA plasmid were linearized by AgeI and *Dra* I respectively, and purified by MinElute PCR Purification Kit (Qiagen, Duesseldorf, Germany). MMESSAGE mMACHINE T7 Ultra Kit (Ambion, TX, USA) were used to produce Cas9 mRNA. MEGA Shortscript and Clear Kit (Ambion, TX, USA) were used to produce and purify the sgRNA. Wild-type C57BL/6 superovulated females were mated with C57BL/6 males to obtain zygotes for Cas9 mRNA and sgRNA injection.

### Genotyping

Edited founders with *Hspa1l* frameshift mutations were mated with *Hspa1l*^+∕+^ for at least three generations to avoid off-targets. The genotype identification of their offspring was completed by PCR amplification (primers: Forward, 5′-TACTGACGAAGATGAAGGAGACT-3′; Reverse, 5′-CGCTTGTTCTGGCTGATGTC-3′) and Sanger sequencing. The results of sequencing were analyzed by SnapGene (version 3.2.1).

### Heat treatment

Mice used for heat treatment were anaesthetized by 1.2% Avertin (Sigma-Aldrich, St. Louis, USA), and lower half of the body of adult (8–10 weeks) *Hspa1l*^−∕−^ and*Hspa1l*^+∕+^ male mice were subjected to water bath for 30 min at 42 °C after narcotization. Animals were dried and returned to their cages for another 4 h. Mice narcotized and left in their cages at normal temperature were used as controls. All mice were euthanized for further analyses after the treatment (*n* = 5).

### Western blot

Testicular protein (*n* = 5 per group) was extracted by RIPA buffer (P0013C, Beyotime, Shanghai, China) containing protease inhibitor cocktail (B14002, Bimake, Houston, USA). The Bradford method was used to measure the concentration of protein in each sample. Samples were separated by SDS-PAGE of 10% acrylamide gel and electrotransferred to a PVDF membrane. The membranes were then blocked with 5% skim milk in TBS for 1 h at room temperature and followed by 2 h incubation with the primary rabbit anti-HSPA1L (1:1500 dilution, 13970-1, Proteintech, PA, USA) and anti-GAPDH (1:1500 dilution, 14C10, Cell Signaling Technology, MA, USA) at room temperature. After three washes with TBST, the membranes were incubated with secondary antibodies (1:2000 dilution, 31,460, Thermo Fisher, NY, USA) for 40 min at room temperature. ChemiDoc XRS+ System (Bio-Rad, CA, USA) were used for visualizing specific proteins bands.

### Histology

The tissues from mouse testes or epididymis were fixed in modified Davidson’s fluid overnight, and stored in 70% ethanol. This was followed by a series of ethanol dehydration steps. Finally, the tissue was embedded in paraffin. Sections (5 *μ*m) were spread onto slides then dried overnight at 65 °C. Tissue sections were stained with hematoxylin and eosin (H & E) after deparaffination by immersing in serial concentrations of ethanol. Sperm samples obtained from the cauda epididymis were spread onto slides and fixed with 4% paraformaldehyde in PBS for 30 mins, then washed and stained with H&E for histological analysis. Mammalian sperms are considered abnormal when some certain morphology appears, including head abnormal and tail abnormal. Head abnormal includes a hairpin at the neck, headless, and a hammer like, collapsed, triangular or thin elongated head. Absent, short, angular or irregular tails are assessed as tails abnormal (Gatimel et al., 2017; Kawai et al., 2006). All observations were made using bright field microscopy (ZEISS Axio Skop Plus2, Jena, Germany).

### Immunofluorescence and TUNEL assay

Testis sections were rehydrated and antigens were retrieved in sodium citrate buffer by boiling for 10 min. 5% BSA was used to block the sections at room temperature for 2 h after cooled. Incubation with primary antibodies (list in  Table S1) were done overnight at 4 °C. The membranes were then washes three times with PBST (0.02% Tween 20 in PBS) before incubation with secondary antibody (list in  Table S1) and Hoechst 33342 (1:1000 dilution, Invitrogen, CA, USA) at 37 °C for 1 h. Sections were then washed and mounted. Terminal deoxynucleotidyl transferase mediated nick-end labeling (TUNEL) assay (Vazyme, Nanjing, China) was used to detected apoptotic cells in testis according to the manufacturer’s protocols (*n* = 5 per group). LSM800 confocal microscope (Carl Zeiss AG, Jena, Germany) was used for Images capture.

### Fertility test

Adult *Hspa1l*^−∕−^ and *Hspa1l*^+∕+^ male mice were bred with *Hspa1l*^+∕+^ C57BL/6 females, respectively, and *Hspa1l*^−∕−^ female mice were also bred with *Hspa1l*^+∕+^ C57BL/6 males to carry out fertility tests. The mice were checked every morning for a vaginal plug and the date and number of pups were recorded from each litter (*n* = 5).

### Epididymal sperm analysis

Several cuts were made throughout the cauda epididymis and suspended in 10% FBS in modified HTF medium (FUJIFILM Irvine Scientific, CA, USA) at 37 °C for 5 min to obtain mature sperm. 10 µl sperm samples were analyzed using computer assisted semen analysis (Hamilton Thorne Research, MA, USA). The motility, progressive motility, and sperm concentration of the Hspa1l^−∕−^ mice and controls were then measured and analyzed (*n* = 5).

### Quantitative RT-PCR

Total RNA of tissues (*n* = 5 per group) was extracted by TRIzol reagent (Invitrogen, CA, USA). NanoDrop 2000C (Thermo, MA, USA) was used to determine the RNA concentration. PrimeScript RT Master Mix (Takara, CA, USA) was used for reverse transcription of 500 ng total RNA according to the standard protocol. The cDNA was diluted (1:4) and detected by quantitative RT-PCR using a AceQ qPCR SYBR Green Master Mix (Vazyme, Nanjing, China) set to the following conditions: 95 °C denatured for 5 min, 95 °C denatured for 10 s for 40 amplification cycles and annealing and extension at 60 °C for 30 s. 18s rRNAwas the positive control used for normalizing the gene expression and the sequences of primers used in this paper are listed in Table S2.

### Statistical analysis

All data/experiments were repeated at least 5 times, and were represented as the mean ± SD. Independent Student’s  *t*-test or one-way ANOVA were used for statistic comparison.  *P*-value <0.05 was considered statistically significant. GraphPad Prism 6.02 was used to analyze data and draw graphs.

## Results

### Expression analysis of Hspa1l in mice

**Figure 1:**
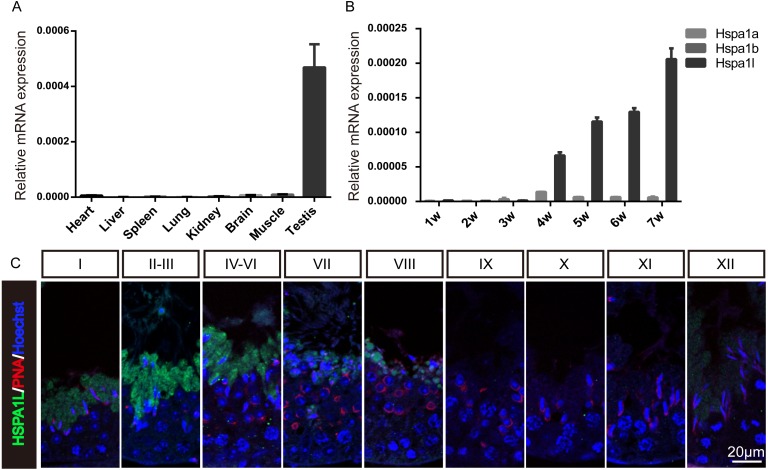
Expression of *Hspa1l* in mouse. (A) Expression analysis of *hspa1l* in mouse tissues, including heart, liver, spleen, lung, kidney, brain, muscle and testis by RT-qPCR. (B) Expression of *hspa1a*, *hspa1b* and* hspa1l* in mouse testes at different developmental stages, including 1, 2, 3, 4, 5, 6 and 7 weeks by RT-qPCR. 18s served as a cDNA loading control in (A–B). (C) HSPA1L staining at specific developmental stages of the mouse male germ cells by immunofluorescence. PNA, shows spermatids.

To investigate the expression of *Hspa1l* in mice, multi-tissue expression analysis were used, which showed that *Hspa1l* was predominantly expressed in testis (Fig. 1A and Fig. S1). The *Hspa1l* mRNA levels start to build up in the testis of 4 weeks old mice pups followed by high expression levels as they grow (Fig. 1B). It is the most abundant of the 3 genes from the MHC III cluster in 17B1 in adult testis, significantly higher than *Hspa1a* and *Hspa1b*. Immunofluorescence analysis showed that HSPA1L was expressed only in spermatids in testes, as it was weakly detectable from step 12 spermatids of stage I, and was the strongest expressed in step 14 and 15 spermatids at stages II–VI (Fig. 1C).

### Generation of Hspa1l^−∕−^ mice

**Figure 2:**
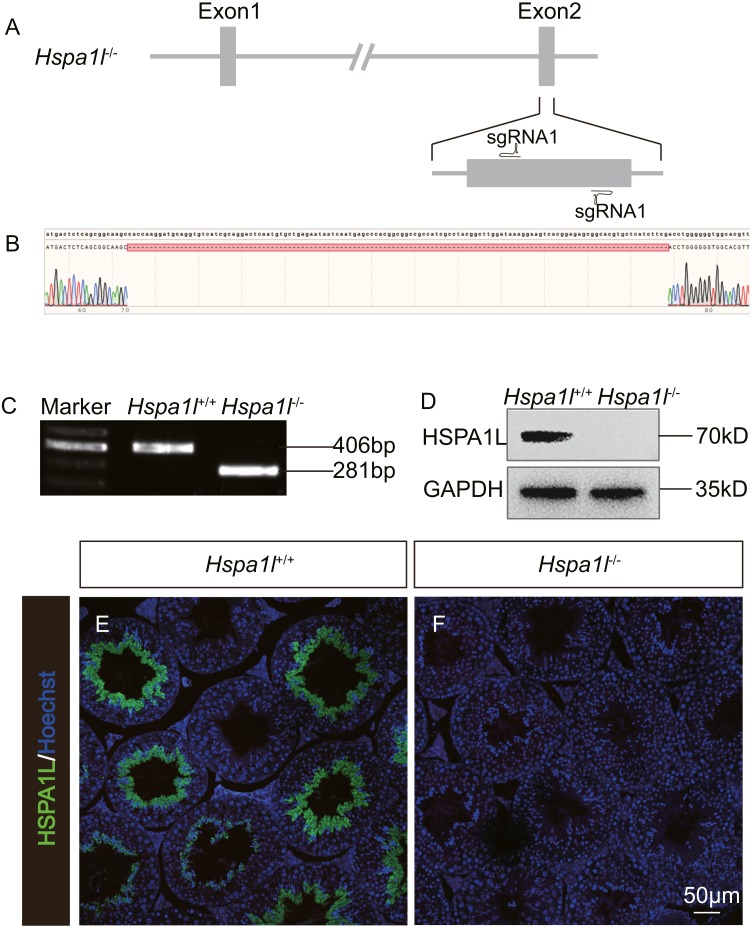
Generation of *Hspa1l*^−∕−^ mice. (A) Schematic representation of targeting strategy using CRISPR/Cas9 system; (B) Sanger sequencing shows a 125-bp deletion were detected in* Hspa1l*^−∕−^ mice; (C) Agarose gel electrophoresis analysis proved the 125-bp deletion in *Hspa1l*^−∕−^ mice; (D) Western blot showing no band at expected size (70 kD) in *Hspa1l*^−∕−^ testis; (E–F) HSPA1L expression from *Hspa1l*^+∕+^ and *Hspa1l*^−∕−^ adult mice by immunofluorescence.

In order to investigate the function of *Hspa1l* in testis, we generated a *Hspa1l* mutant mouse model using the CRISPR/Cas9 system. We created a frameshift by deleting 125 base pairs (bp) of exon 2 of *Hspa1l* (Fig. 2A). The resultant deletion was confirmed by PCR and Sanger sequencing (Figs. 2B and 2C). Western blot and immunofluorescence analyses showed that HSPA1L was undetectable in *Hspa1l*^−∕−^ homozygote testis (Figs. 2D–2F). All *Hspa1l* ^−∕−^ mice showed normal development and were viable.

**Figure 3:**
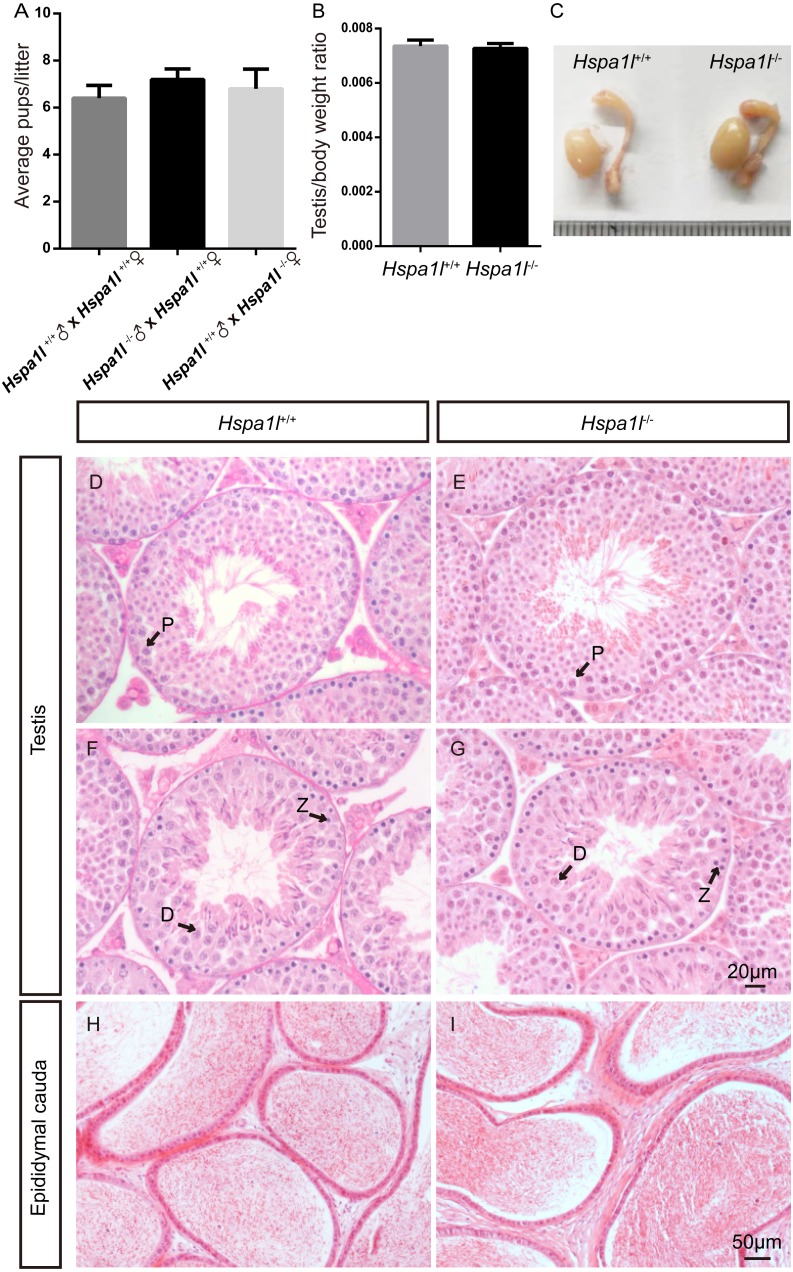
*Hspa1l*^−∕−^ mice showed normal spermatogenesis. (A) Fertility tests show average pups per litter of *Hspa1l*^+∕+^ and* Hspa1l*^−∕−^ mice, *n* = 5. (B) Average testis weight/body weight of *Hspa1l*^+∕+^ and* Hspa1l*^−∕−^ male mice, *n* = 5; (C) Morphology of *Hspa1l*^+∕+^ and *Hspa1l*^−∕−^ adult mice in testis and epididymis; (D–I) H&E-stained sections of testes and epididymal cauda from *Hspa1l*^+∕+^ and *Hspa1l*^−∕−^ mice; P, pachytene; Z, zygotene; D, diplotene.

### Hspa1l^−∕−^ mice are fertile with normal spermatogenesis

**Figure 4:**
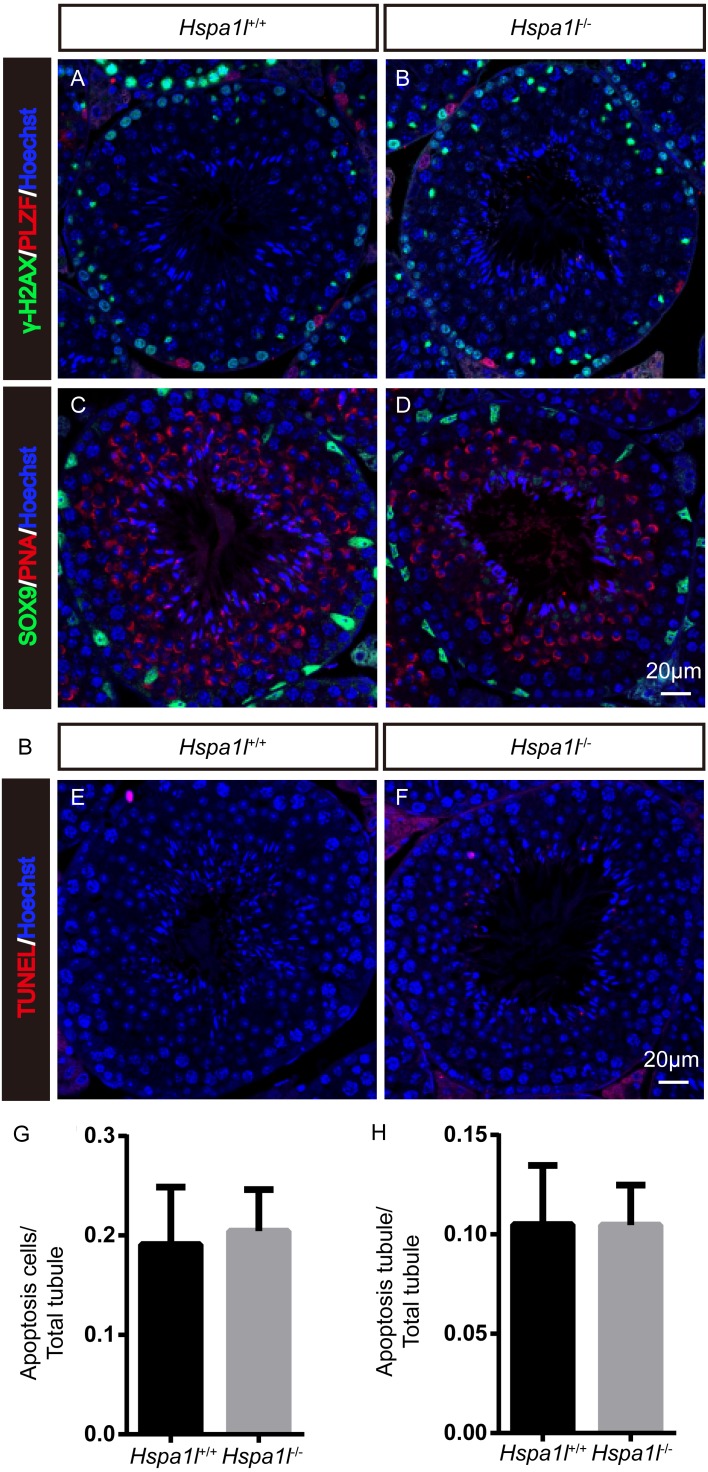
Immunofluorescence and apoptosis analysis of *Hspa1l*^+∕+^ and *Hspa1l*^−∕−^ testes. (A–D) The PLZF, *γ*-H2AX, PNA and Sox9 signals shows the locations of spermatogonia, spermatocytes, spermatids and Sertoli cells in testis sections from both *Hspa1l*^+∕+^ and *Hspa1l*^−∕−^ mice, respectively. (E–F) Testicular sections of TUNEL assay in *Hspa1l*^+∕+^ and *Hspa1l*^−∕−^ mice; (G) Average apoptotic cells per seminiferous tubule in *Hspa1l*^+∕+^ and *Hspa1l*^−∕−^ testicular sections; (H) Average apoptotic cells per seminiferous tubules in *Hspa1l*^+∕+^ and *Hspa1l*^−∕−^ testicular sections, *n* = 5.

Unlike the *Hspa2* mutant mice (Dix et al., 1996), *Hspa1l*^−∕−^ males were fertile (*n* = 5, *P* = 0.344) (Fig. 3A). Intercrossed *Hspa1l*^+∕−^ mice offspring show normal litter size and sex ratios under the expected Mendelian distribution. There was no difference in size of testes (*n* = 5, *P* = 0.262) and epididymides between *Hspa1l*^−∕−^ and *Hspa1l*^+∕+^ male mice (Figs. 3B and 3C). In addition, normal spermatogenic cells of all stages were observed in the seminiferous tubules of adult *Hspa1l*^−∕−^ mice by H&E staining (Figs. 3D–3I). Immunofluorescence analysis showed the presence of normal PLZF-positive spermatogonia, *γ*-H2AX-positive spermatocytes, and PNA-positive acrosomes in spermatids (Figs. 4A–4D). TUNEL analysis of testicular sections showed that the number of apoptotic cells per tubule (*n* = 5, *P* = 0.687) and the ratio of apoptotic tubules (*n* = 5, *P* = 0.986) were similar between *Hspa1l*^−∕−^ and *Hspa1l*^+∕+^ control testes (Figs. 4E–4H).

### Spermatozoa are normal in Hspa1l^−∕−^ mice

**Figure 5:**
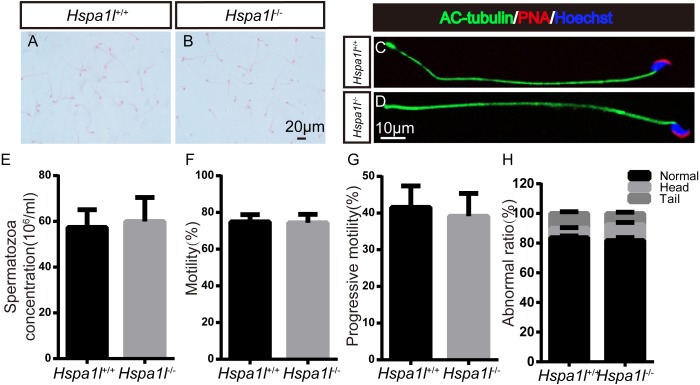
Spermatozoa appear normal in *Hspa1l*^−∕−^ mice. (A–B) H&E-stained spermatozoa from *Hspa1l* ^+∕+^ and *Hspa1l*^−∕−^ mice; (C–D) AC-tubulin, PNA signals from *Hspa1l*^+∕+^ and *Hspa1l*^−∕−^ spermatozoa by immunofluorescence; (E) Sperm concentration of the cauda epididymal from *Hspa1l*^+∕+^ and *Hspa1l*^−∕−^ mice, *n* = 5; (F) Average rate of motile sperm and (G) progressive sperm from *Hspa1l*^+∕+^ and *Hspa1l*^−∕−^ mice, *n* = 5; (H) Abnormal epididymal sperm count from *Hspa1l*^+∕+^ and* Hspa1l*^−∕−^ mice, *n* = 5.

There was no significant difference between the whole epididymal sperm count (*n* = 5, *P* = 0.669) and the morphology of spermatozoa from the epididymal cauda between *Hspa1l*^−∕−^ and *Hspa1l*^+∕+^ mice (Figs. 5A–5E). The sperm motility (*n* = 5, *P* = 0.842) and progressive motility (*n* = 5, *P* = 0.546) in *Hspa1l*^−∕−^ mice were comparable to the *Hspa1l*^+∕+^ controls. Morphological analysis also showed the ratio of normal sperm (*n* = 5, *P* = 0.445) were unaffected in *Hspa1l*^−∕−^ male mice (Figs. 5F–5H). These results show that the deletion of *Hspa1l* does not affect spermatogenesis and fertility in *Hspa1l*^−∕−^ mice.

### Hspa1l is not essential in responses to heat stress

**Figure 6:**
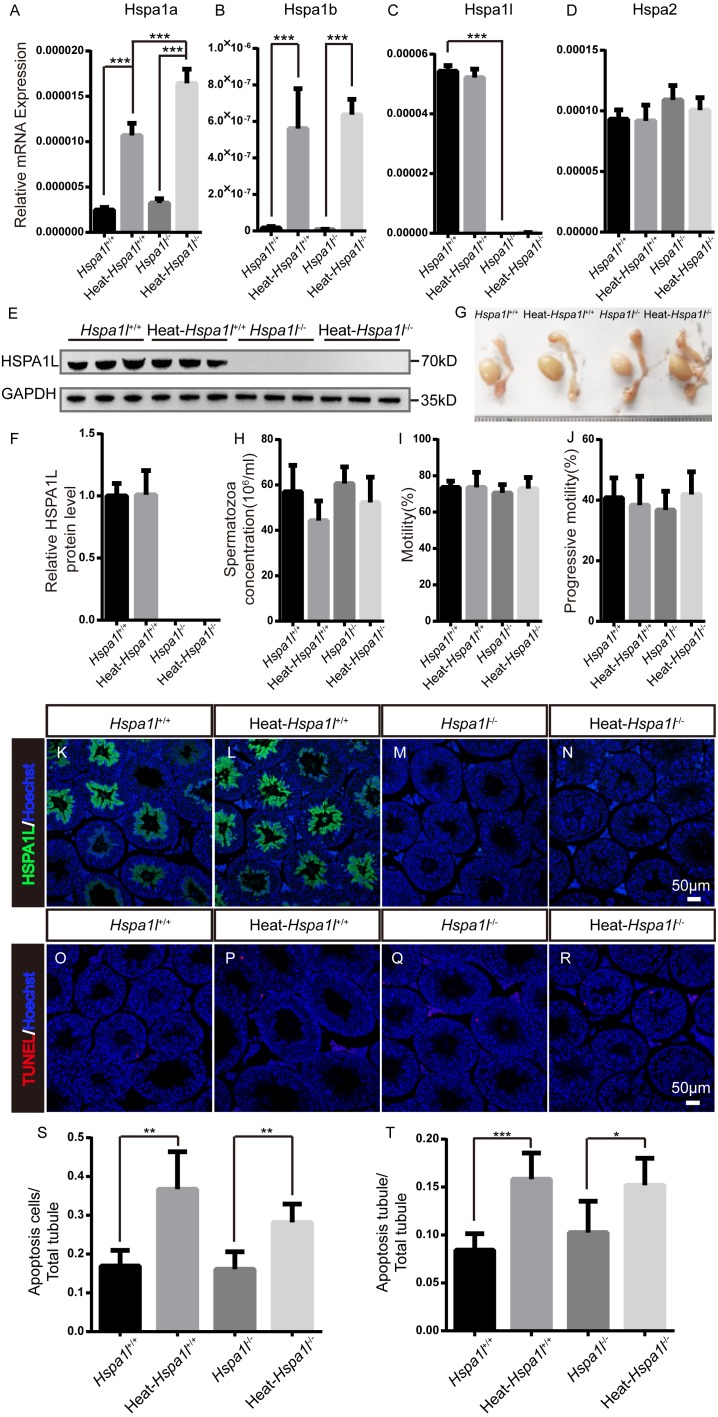
Analysis of testis and sperm in *Hspa1l*^−∕−^ mice after heat stress. (A–D) Expression of *Hspa1a*, *Hspa1b*, *Hspa1l* and *Hspa2* in *Hspa1l*^+∕+^ and *Hspa1l*^−∕−^ mice testis with or without heat treatment, *n* = 5, *** *P* < 0.001; (E) HSPA1L expression in *Hspa1l*^+∕+^ and *Hspa1l*^−∕−^ mice testis with or without heat treatment by western blot and (F) quantitated by densitometric analysis, *n* = 5; (G) Testes and epididymides from *Hspa1l*^+∕+^ and *Hspa1l*^−∕−^ adult mice with or without heat treatment; (H) Cauda epididymal sperm concentration from *Hspa1l*^+∕+^ and *Hspa1l*^−∕−^ mice with or without heat treatment, *n* = 5; (I) Average sperm motility and (J) progressive motility from *Hspa1l*^+∕+^ and *Hspa1l*^−∕−^ mice with or without heat treatment, *n* = 5; (K–N) HSPA1L staining in *Hspa1l* ^+∕+^ and *Hspa1l*^−∕−^ mice testis with or without heat treatment by immunofluorescence; (O–R) TUNEL assay of *Hspa1l*^+∕+^ and *Hspa1l* ^−∕−^ testes with or without heat treatment; (S) Average apoptotic cells per seminiferous tubule in testicular sections, *n* = 5, ** *P* < 0.01; (T) Average apoptotic cells per seminiferous tubules in testicular sections, *n* = 5. * *P* < 0.05, *** *P* < 0.001.

HSP70s are strong anti-apoptotic proteins which can prevent apoptosis from different stresses, such as heat shock (Durairajanayagam, Agarwal & Ong, 2015), oxidative stress (Ikwegbue et al., 2017), infection (Mays et al., 2019), etc. We applied heat stress to test the anti-apoptotic ability of HSPA1L in testis. We found that *Hspa1a* and *Hspa1b* mRNA levels increased after heat treatment, while those of *Hspa1l* and *Hspa2* were not affected (Figs. 6A–6D). We measured the expression of other *Hsp70s*, and found that *Hspa5* is down-regulated after heat stress. However, *Hspa9* and *Hspa12b* were downregulated only in heat treated *Hspa1l*^−∕−^ testes (Fig. S2). Further analysis of protein expression levels showed that HSPA1L remained unaffected under heat stress (*n* = 5, *P* = 0.925), consistent with its RNA levels (Figs. 6E–6F and Fig. S3). Furthermore, compared to the *Hspa1l*^+∕+^ control, there was no significant differences in testes and epididymides size (Fig. 6G), sperm count (*n* = 5, *P* = 0.091), sperm motility (*n* = 5, *P* = 0.809) and progressive motility (*n* = 5, *P* = 0.708) with or without heat stress in *Hspa1l*^−∕−^ (Figs. 6H–6J). TUNEL analysis of testicular sections showed no difference between the *Hspa1l*^−∕−^ and control testes after heat stress for both the number of apoptotic cells (*n* = 5, *P* = 0.110) and the number of apoptotic tubules (*n* = 5, *P* = 0.312). *Hspa1l*^−∕−^ testis responded to heat stress in a way comparable to controls (Figs. 6K–6T). Thus, *Hspa1l* doesn’t play an important role during spermatogenesis or in the anti-apoptotic response to stress, as suggested in the earlier reports.

## Discussion

In this study, we examined the possible function of *Hspa1l*, a member of *Hsp70* family of proteins, with testis-predominant expression. *Hspa1l* showed developmental stage-specific expression pattern during testis development. It typically starts to express from step 12 spermatids of stage I spermatogenesis. Previously, it was thought to have important functions in spermatogenesis and was therefore associated with an increased risk of idiopathic male infertility (Kohan, Tabiee & Sepahi, 2019). However, our studies demonstrate that *Hspa1l*^−∕−^ *Hspa1l*^−∕−^ mice have normal spermatogenesis with appearance of all types and stages of spermatogenic cells. Both male and female *Hspa1l*^−∕−^ mice were fertile without any apparent developmental abnormalities.

HSPA1A/B, two members of HSP70 family from the same cluster as HSPA1L, are normally located in cytosol, and translocate to the nucleus rapidly under emergency circumstances, such as heat stress, to protect the chromatin (Lindquist & Craig, 1988). HSPA1L is highly expressed in testis, and located in cytosol as well. Testis is susceptible to hyperthermia, which could damage spermatogenesis and lead to male infertility (Durairajanayagam, Agarwal & Ong, 2015). We found that while *Hspa1l* is a testis-predominant gene, it is not essential to spermatogenesis. Therefore, we investigated whether it might play an important role in heat stress-induced responses. However, unlike HSPA1A/B, HSPA1L did not translocate to the nucleus after heat stress, indicating that although these three proteins are highly analogous, the function of HSPA1L may be very different from HSPA1A/B.

Cells enhance their expression of stress- inducible HSP70s to respond to environmental stresses (Chen, Feder & Kang, 2018a; Ikwegbue et al., 2017; Radons, 2016; Singh et al., 2010). HSPA1L was thought to have the ability to enhance cell survival in testes following multiple stresses, such as a heat stress. By that reasoning, the *Hspa1l*^−∕−^ testis may be more sensitive to heat stress. Also, the expression of HSPA1L is focused in spermatids, so it was reasonable to speculate that heat treated *Hspa1l*^−∕−^ testis might show an increase of apoptotic spermatids. However, TUNEL analysis of *Hspa1l*^−∕−^ testicular sections showed that no increase in the number of apoptotic cells compared to the controls after the heat treatment. Thus, the deletion of *Hspa1l* does not affect the germ cells’ response to heat stress.

It has been reported that *Hspa1l* mutants have increased risk of male infertility (Huusko et al., 2018; Takahashi et al., 2017), but our studies show that there was no difference in the fertility of *Hspa1l*^−∕−^ mutant mice compared to *Hspa1l*^+∕+^ controls. Analysis of the expression levels of other *Hsp70s* showed no up-regulation in *Hspa1l*^−∕−^ testes. However, the expression levels of *Hspa1a* and *Hspa1b* increased in *Hspa1l*^−∕−^ testes compared with *Hspa1l*^+∕+^ testis after heat treatment, which implied that *Hspa1a* and *Hspa1b* possibly compensated for *Hspa1l* function under heat stress. Interestingly, we found that *Hspa5* was downregulated in *Hspa1l*^−∕−^ testes, and *Hspa9* and *Hspa12b* were downregulated in *Hspa1l* ^−∕−^ testes after heat stress compared to *Hspa1l*^+∕+^. The expression of these *Hsp70* family members were affected by *Hspa1l* expression, the biological significance of which needs to be examined further.

As we investigated that heat stress does not affect cells survival in *Hspa1l*^−∕−^ mutants, the affects from other stresses have not yet been studied. Especially, the study of inflammation in *Hspa1l*^−∕−^ mutants because *Hspa1l* and *Hspa1a/b* all fall under the MHC class III family. Testis is a tissue with immune privilege thanks to the blood-testis barrier (BTB) (Fijak, Bhushan & Meinhardt, 2011). Considering the different expression pattern of *Hspa1l* from *Hspa1a/b*, it indicated that *Hspa1l* may has a crucial role in testes when encounter inflammation, which needs further investigation.

## Conclusions

*Hspa1l* is highly and constitutively expressed in testis. However, *Hspa1l*^−∕−^ mice are fertile, and showed no abnormality in spermatogenesis. Further, heat stress does not exacerbate the germ cells’ apoptosis in *Hspa1l*^−∕−^ testes. This suggests that there is no essential function of *Hspa1l* in response to heat stress, possibly compensated by other members of the family, *Hspa1a* and *Hspa1b.* The *in vivo* functional studies of HSPA1L could provide a basis for further elucidation of functions of the highly conserved HSP70s family.

##  Supplemental Information

10.7717/peerj.8702/supp-1 Figure S1 Expression analysis of HSPA1L in mouse tissues, including heart, liver, spleen, lung, kidney, brain, muscle and testis by western blot

10.7717/peerj.8702/supp-2 Figure S2 Expression of *Hspa5*, *Hspa8*, *Hspa9*,* Hspa12a*, * Hspa12b* and *Hspa13* in *Hspa1l*^+∕+^ and *Hspa1l*^−∕−^ mice testis with or without heat treatment *n* = 5, * *P* < 0.05, *** *P* < 0.001.

10.7717/peerj.8702/supp-3 Figure S3 HSPA1L expression in *Hspa1l*^+∕+^ and *Hspa1l*^−∕−^ mice testis with or without heat treatment by western blot, the other two supplements of Fig. 6B.

10.7717/peerj.8702/supp-4 Data S1 Raw data for mRNA expression in tissues

10.7717/peerj.8702/supp-5 Data S2 Raw data for mRNA expression in development stages

10.7717/peerj.8702/supp-6 Data S3 Raw data for fertility test

10.7717/peerj.8702/supp-7 Data S4 Raw data for testis-body weight

10.7717/peerj.8702/supp-8 Data S5 Raw data for apoptic positive cell ratio

10.7717/peerj.8702/supp-9 Data S6 Raw data for spermatozoa concentration and motility

10.7717/peerj.8702/supp-10 Data S7 Raw data for spermatozoa abnormality ratio

10.7717/peerj.8702/supp-11 Data S8 Raw data for mRNA expression of Hspa1a, Hspa1b, Hspa1l and Hspa1l

10.7717/peerj.8702/supp-12 Data S9 Raw data for densitometric analysis of western blot

10.7717/peerj.8702/supp-13 Data S10 Raw data for spermatozoa concentration and motility with heat treatment

10.7717/peerj.8702/supp-14 Data S11 Raw data for apoptic positive cell ratio with heat treatment

10.7717/peerj.8702/supp-15 Data S12 Raw data for mRNA expression of hspa5, hspa8, hspa9, hspa12a, hspa12b and hspa13

10.7717/peerj.8702/supp-16 Data S13 Raw data for PCR of Figure 2C

10.7717/peerj.8702/supp-17 Data S14 Raw data for WB of Figure 2D

10.7717/peerj.8702/supp-18 Data S15 Raw data for WB of Figure 6B

10.7717/peerj.8702/supp-19 Data S16 Raw data for WB of Figure S1

10.7717/peerj.8702/supp-20 Data S17 Raw data for WB of Figure S3

10.7717/peerj.8702/supp-21 Table S1 List of antibodies

10.7717/peerj.8702/supp-22 Table S2 Primer sequences

## Additional Information and Declarations

### Data Availability

The following information was supplied regarding data availability: The raw measurements are available in the Supplementary Files.
