# Supplementary figures and images for: The heat shock protein family gene Hspa1l in male mice is dispensable for fertility
Source: PeerJ. 2020 Mar 23;8:e8702. doi: 10.7717/peerj.8702 (PMC7098389; doi:10.7717/peerj.8702)

Heart Liver Spleen Lung Kidney Brain Muscle Testis

HSPA1L

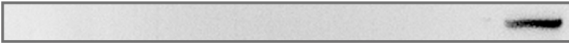

—70kD

GAPDH

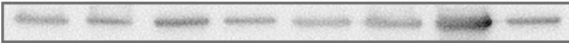

—35kD

Supplement: Figure S1 [file peerj-08-8702-s001.pdf]

Hspa5

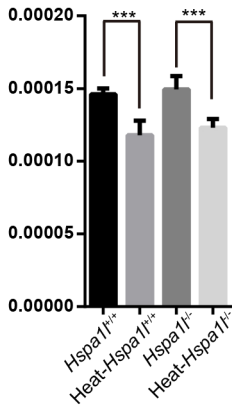

Hspa8

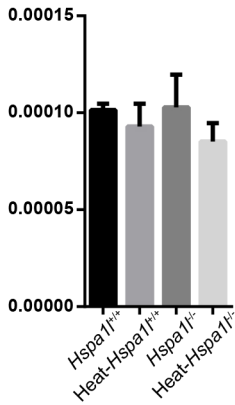

Hspa9

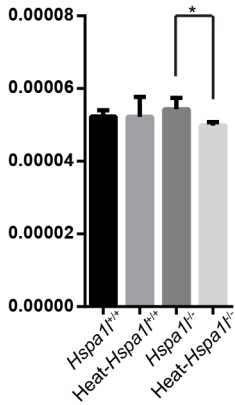

Hspa12a

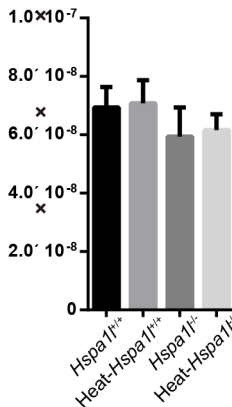

Hspa12b

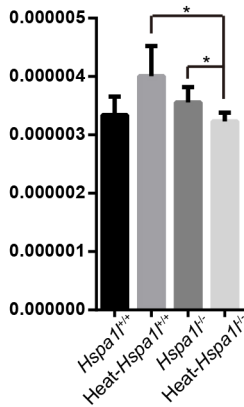

Hspa13

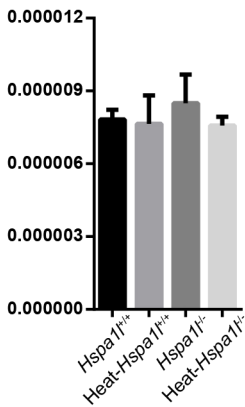

Supplement: Figure S2 — n = 5, * P < 0.05, *** P < 0.001. [file peerj-08-8702-s002.pdf]

*Hspa1l*<sup>+/+</sup> Heat-*Hspa1l*<sup>+/+</sup> *Hspa1l*<sup>-/-</sup> Heat-*Hspa1l*<sup>-/-</sup>

4

5

4

5

4

5

4

5

HSPA1L

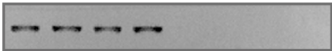

— 70kD

GAPDH

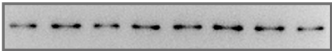

— 35kD

Supplement: Figure S3 [file peerj-08-8702-s003.pdf]

Marker WT HOM —

406bp—  
281bp—

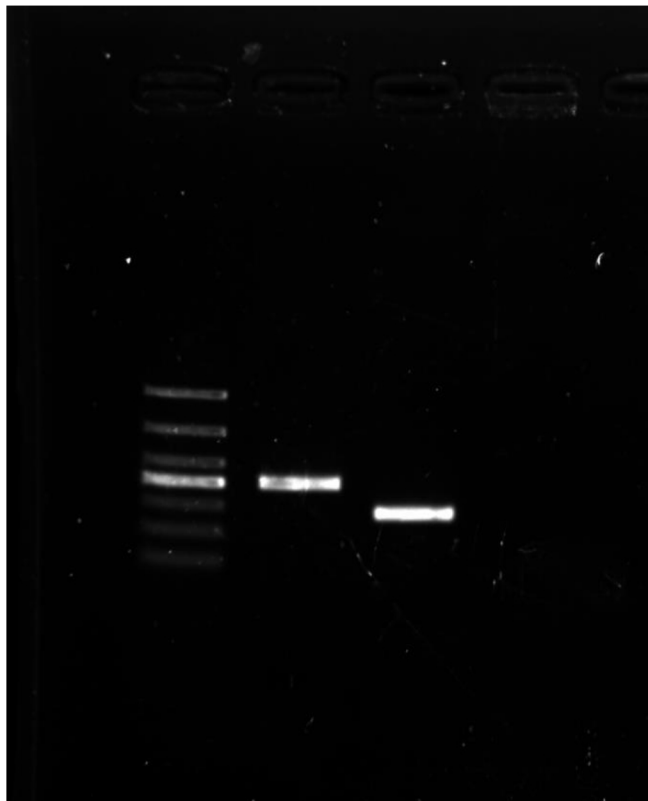

Supplement: Data S13 [file peerj-08-8702-s016.pdf]

PVDF

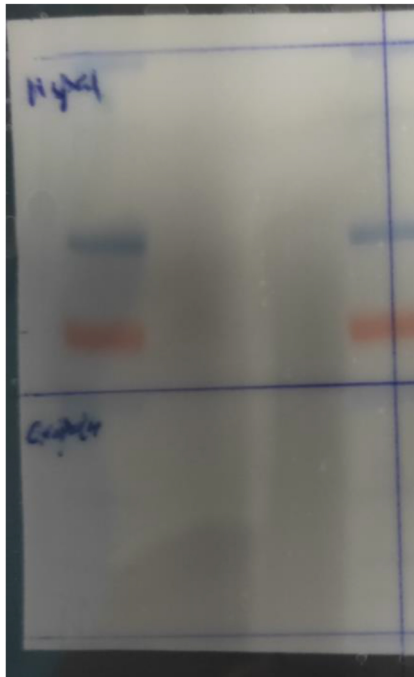

Marker WT HOM

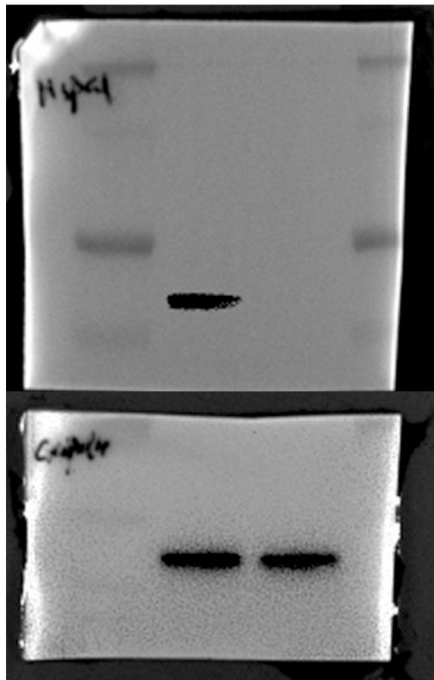

— HSPA1L

— GAPDH

Supplement: Data S14 [file peerj-08-8702-s017.pdf]

PVDF

Marker    WT    Heat-WT    HOM    Heat-HOM  
          1 2 3    1 2 3    1 2 3    1 2 3

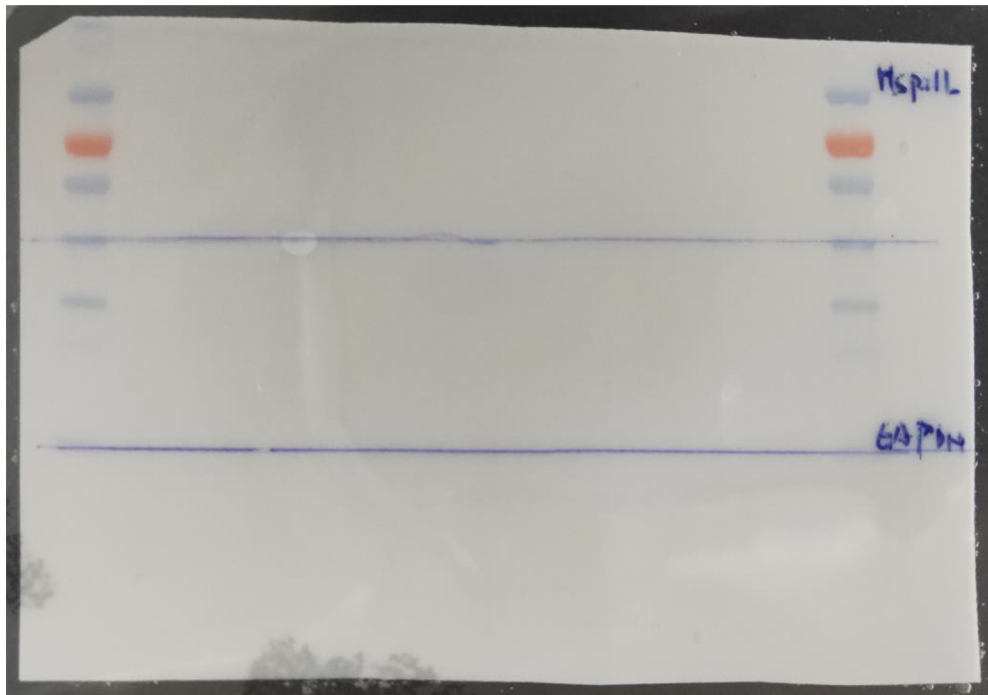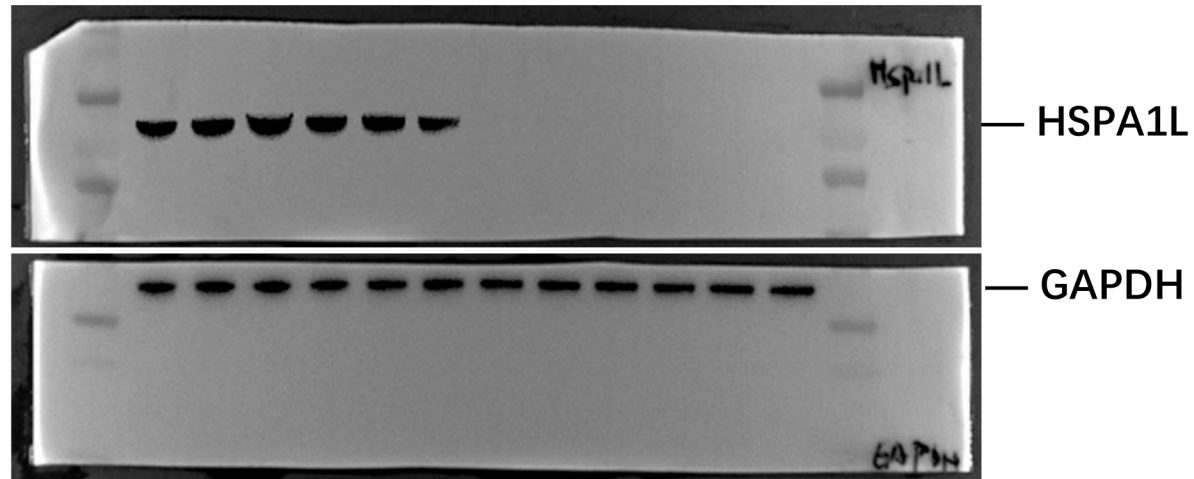

Supplement: Data S15 [file peerj-08-8702-s018.pdf]

# PVDF

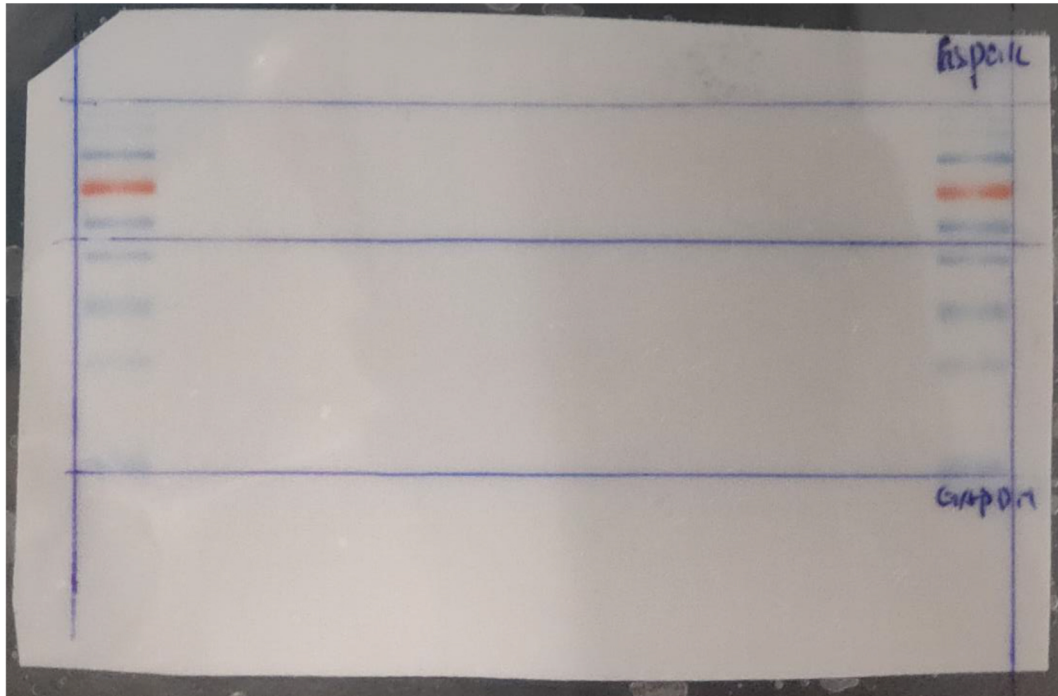

Marker Heart Liver Spleen Lung Kidney Brain Muscle Testis Marker

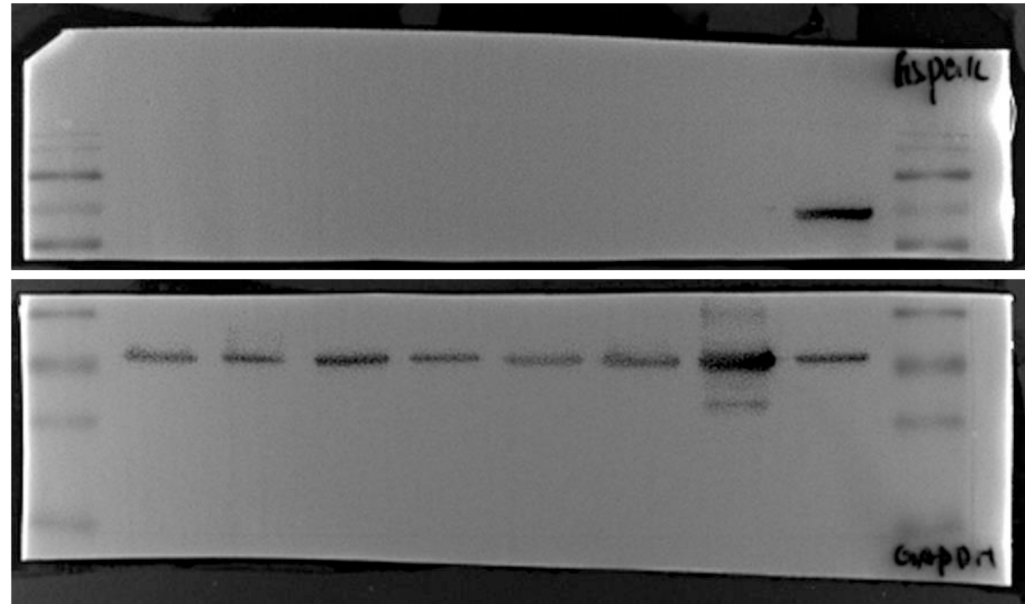

— HSPA1L

— GAPDH

Supplement: Data S16 [file peerj-08-8702-s019.pdf]

PVDF

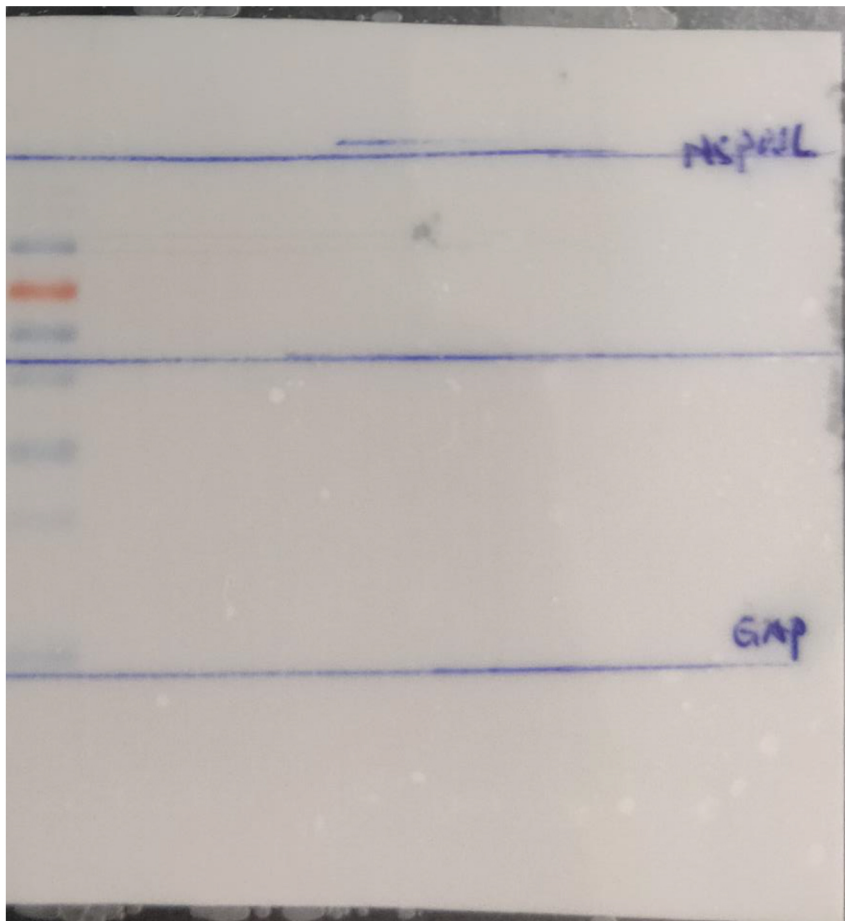

| Marker | WT |   | Heat-WT |   | HOM |   | Heat-HOM |   |
|--------|----|---|---------|---|-----|---|----------|---|
|        | 4  | 5 | 4       | 5 | 4   | 5 | 4        | 5 |

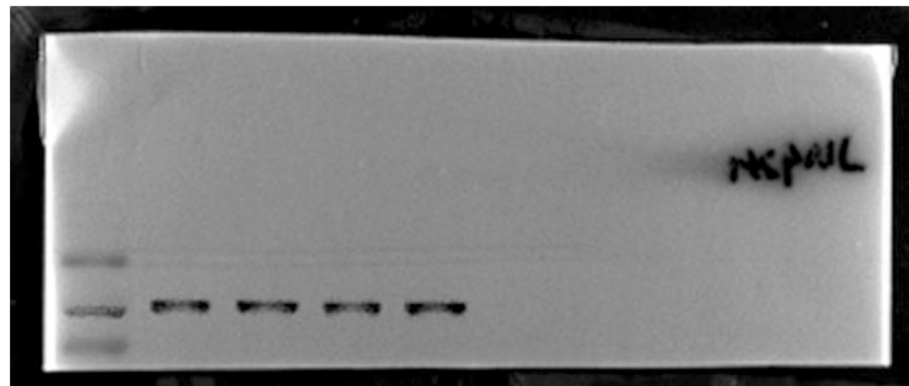

— HSPA1L

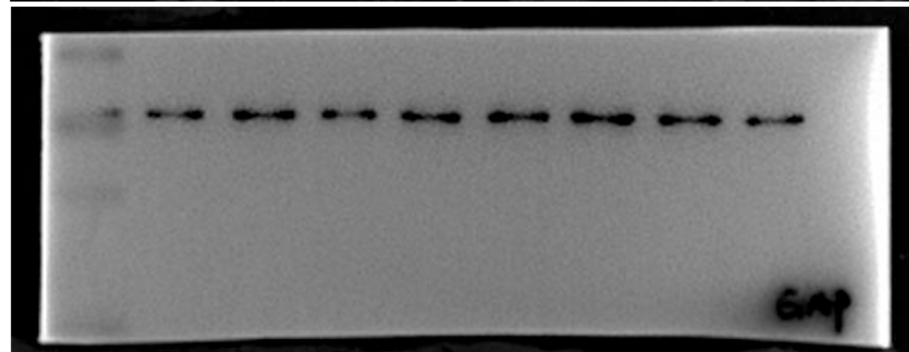

— GAPDH

Supplement: Data S17 [file peerj-08-8702-s020.pdf]
